# Untargeted Metabolomics Reveals Novel Biomarkers and Pathways for Early Detection and Pathogenesis of Alzheimer's Disease

**DOI:** 10.1002/alz70856_097300

**Published:** 2025-12-24

**Authors:** Prabhakar Tiwari

**Affiliations:** ^1^ Department of Anatomy, AIIMS, New Delhi‐110029, India, New Delhi, New Delhi, India

## Abstract

**Background:**

Alzheimer's disease (AD) is a progressive neurodegenerative disorder characterized by cognitive decline, amyloid‐beta (Aβ) plaques, and tau tangles. Early diagnosis is vital for timely intervention. This study investigates metabolic alterations in AD and their associations with plasma biomarkers to uncover potential early diagnostic markers and elucidate disease mechanisms.

**Methods:**

A case‐control study was conducted involving 25 AD patients and 25 healthy controls (HC). Cognitive function was assessed using the Clinical Dementia Rating (CDR) scale, and depression was evaluated with PHQ‐9. Untargeted metabolomics profiling using LC‐MS identified key metabolites, analyzed via MetaboAnalyst (v6.0). Plasma biomarkers, including Aβ40, Aβ42, *p*‐tau‐181, *p*‐tau‐217, NFL, APOE4, CRP, BDNF, and oxidative stress markers, were quantified using ELISA. Statistical analyses, including PCA, volcano plots, ROC curves, and Pearson correlations, were applied to assess diagnostic utility and biomarker associations.

**Results:**

AD patients exhibited significant cognitive impairment (CDR‐G1: 1.44 ± 0.64) and depressive symptoms (PHQ‐9: 4.76 ± 4.15) compared to HC. Metabolomics identified 20 significantly altered metabolites, with leucine (AUC: 0.7632), ascorbic acid (AUC: 0.7472), and octadecanedicarboxylic acid (AUC: 0.9104) showing high diagnostic potential. Elevated leucine levels were strongly correlated with memory deficits (*r* = ‐0.72, *p* < 0.01), while ascorbic acid was inversely associated with neuroinflammatory markers (e.g., IL‐6; r = ‐0.65, *p* < 0.05).

**Conclusion:**

This study identifies distinct metabolic signatures and plasma biomarkers with diagnostic and therapeutic potential for AD. Leucine and ascorbic acid emerge as promising markers linked to cognitive decline and neuroinflammation, respectively, paving the way for early detection and targeted interventions.

